# Genetic Diversity Analysis of the Red Swamp Crayfish *Procambarus clarkii* in Three Cultured Populations Based on Microsatellite Markers

**DOI:** 10.3390/ani13111881

**Published:** 2023-06-05

**Authors:** Jiaqing Liu, Yunfei Sun, Qianqian Chen, Miaomiao Wang, Qin Li, Wenzong Zhou, Yongxu Cheng

**Affiliations:** 1 Centre for Research on Environmental Ecology and Fish Nutrition of the Ministry of Agriculture, Shanghai Ocean University, Shanghai 201306, China; 2 Shanghai Engineering Research Center of Aquaculture, Shanghai Ocean University, Shanghai 201306, China; 3 National Demonstration Centre for Experimental Fisheries Science Education, Shanghai Ocean University, Shanghai 201306, China; 4 Key Laboratory of Integrated Rice-Fish Farming, Freshwater Fisheries Research Center, Chinese Academy of Fishery Sciences, Ministry of Agriculture and Rural Affairs, Wuxi 214081, China; 5 Jiangsu Provincial Aquatic Technology Extension Center, Nanjing 210036, China; 6 Institute of Eco-Environmental Preservation, Shanghai Agricultural Academy of Sciences, Shanghai 201403, China

**Keywords:** *Procambarus clarkii*, microsatellite, genetic diversity, genetic structure

## Abstract

**Simple Summary:**

High-quality seedlings are a prerequisite for healthy aquatic animal breeding. As the scale of *Procambarus clarkii* farming continues to expand, the model of “breeding and catching large and keeping small” has led to serious degradation of the fry. This has restricted the development of the *P. clarkii* industry. Therefore, the selection and breeding of optimal breeds of *P. clarkii* are very important. Investigating intraspecific patterns of genetic diversity may help unravel the species’ genetic structure, evaluate the quality of the fry, and design measures for breeding and conservation. In this study, the genetic diversity of *P. clarkii* from three regions of China was analysed using microsatellite markers. Populations of *P. clarkii* showed high genetic diversity and extensive gene flow. The Xuancheng population could be used as a selective breeding population because it possesses the highest diversity. The results of this study provide a scientific basis for the conservation of germplasm resources and the genetic selection and breeding of *P. clarkii*.

**Abstract:**

With the increasing scale of crayfish breeding, the self-propagation and “catch large and keep small” breeding patterns have led to serious degradation of the fry, so the selection and breeding of high-quality fry is very important. Selecting a population with a high genetic diversity as the base population for breeding can greatly improve the breeding efficiency. Fifteen microsatellite loci were used to understand the genetic structure and diversity of three *Procambarus clarkii* populations in Chongming, Shanghai; Gaoyou, Jiangsu; and Xuancheng, Anhui. The results indicated that the three populations were diverse and the number of alleles, observed heterozygosity, expected heterozygosity, Shannon information index, and polymorphic information content ranged from 4.8 to 6.2, 0.5567 to 0.6257, 0.6166 to 0.7086, 1.1292 to 1.3987, and 0.5446 to 0.6452, respectively. The Xuancheng population had the highest genetic diversity. The genetic differentiation coefficient and gene flow of the three populations were between 0.0553 and 0.1068 and 2.0908 and 4.2708, respectively, and there was extensive genetic exchange between the Chongming and Xuancheng populations. Analyses of molecular variance indicated that the genetic variation was mainly within the population (91.51%) and inter-population genetic variation accounted for 8.49%. The unweighted pair group method with an arithmetic mean clustering map was utilised based on the genetic distance between groups, and the results showed that the Gaoyou group was grouped alone, while the Chongming and Xuancheng groups were clustered together. The structural results indicated that the Chongming and Xuancheng groups had the same origin, although the Xuancheng group possessed a more complex genetic structure. This study indicated that all three populations had a high genetic diversity, with the Xuancheng population exhibiting the highest diversity. The results of the study provide a reference for the selection of base populations in breeding programs and confirm that the Xuancheng population in Anhui has a better genetic background. The selection of the Xuancheng population as one of the base populations for genetic breeding will be more efficient to accumulate superior traits.

## 1. Introduction

The red swamp crayfish *Procambarus clarkii*, commonly known as crayfish and native to northern Mexico and the southern United States, was introduced to China in the 1930s and is now widely distributed there [1]. As an invasive species, it possesses biological characteristics such as fast growth, early maturation, high fecundity, and high adaptability to the environment; therefore, it can adapt quickly to different regional environments and establish different populations [2,3,4]. Based on reports, the total output value of China’s *P. clarkii* industry in 2021 was CNY 422.195 billion, with a farming area of 26 million mu and an output of 2,633,600 tons, and it continues to grow rapidly [5]. However, issues have emerged because of the rapid expansion of farming scale and breeding, in addition to the capture of large and small breeding modes which keeps the young shrimp for breeding and the adult fish for sale. Furthermore, inbreeding can lead to population degradation because offspring of close relatives tend to inherit subtle undesirable traits, seriously affecting the quality of adult shrimp and limiting the development of the crayfish industry. Although genetic diversity is an important factor in crayfish breeding, to our knowledge, there has been no successful breeding of crayfish using genetic data to date.

By analysing the genetic diversity of organisms, it is possible to understand the genetic background and structure of a species and to also assess the quality of its seedlings in order to develop appropriate measures for breeding and conservation [6,7,8].

Microsatellite markers, also known as simple sequence repeats (SSRs), have characteristics of large numbers, wide distribution, hypervariability, codominant inheritance, high conservativeness, and high reproducibility [9,10,11]. SSRs were used in this study to evaluate the genetic status of crayfish, since SSRs have been widely used in animal and plant breeding, evolutionary research, and other fields. In addition, the use of SSR molecular markers for genetic evaluation can improve the breeding efficiency [12,13,14,15,16,17]. Liu et al. [18] used 17 SSR molecular markers to analyse the genetic diversity of 25 different geographic populations of *P. clarkii* in China, indicating that the overall genetic diversity of *P. clarkii* in China is high and the high level of genetic differentiation in the population may be attributed to genetic drift and geographical isolation. Cui et al. [19] used 10 pairs of SSR molecular markers to compare the genetic diversity of the farmed population of *P. clarkii* in Anhui with the wild and farmed populations in Jiangsu and Hubei. Their results showed that the level of genetic diversity of the farmed population of *P. clarkii* in Anhui was higher than that of the wild and farmed populations in Jiangsu and Hubei. Furthermore, it was considered necessary to strengthen the population introgression between different regions to improve the status of inbreeding. Using eight SSR molecular markers, Xing et al. [20] analysed the genetic diversity of *P. clarkii* in eight main production areas in Jiangsu and identified extensive gene exchange and moderate genetic differentiation among the eight populations of *P. clarkii*, suggesting mutual exchange within Jiangsu Province due to anthropogenic factors. These studies demonstrate that SSRs are an appropriate molecular marker tool for analysing the genetic diversity among and within populations of *P. clarkia*. Related studies can reveal the genetic characteristics of different geographical populations of Chinese *P. clarkii* and also lay the foundation for molecularly assisted breeding. This allows scientists to select different breeding populations in a targeted manner according to diversity level and the genetic structure of different populations, helping to reduce effort regarding the selection of baseline breeding populations, improve breeding efficiency, and promote the progress of breeding work.

Selective breeding of *P. clarki* is a hot research topic in China, and the study of genetic diversity is essential for breeding efforts. Current studies have focused on the genetic diversity of wild populations of crayfish, while relatively few studies have been conducted on cultured populations; thus, the main goal of this study was to compare the genetic diversity of cultured populations to determine if they could be used as a base population for breeding. Therefore, 90 muscle samples of *P. clarkii* from three cultured populations were used to analyse the genetic diversity of the *P. clarkii* germplasm using SSR molecular markers in order to compare patterns of genetic diversity among the three populations of *P. clarkii* in Xuancheng, Anhui; Chongming, Shanghai; and Gaoyou, Jiangsu. This study aimed to promote the effective use of germplasm resources and to provide a theoretical basis for quality improvement and molecularly assisted breeding of *P. clarkii*.

## 2. Materials and Methods

### 2.1. Sample Source

In June 2021, *P. clarkii* from Shanghai Chongming (CM), Jiangsu Gaoyou (GY), and Anhui Xuancheng (XC) were collected as the experimental samples. Shanghai samples were collected from the farms of Shanghai Daohong Aquaculture Technology Co., Ltd.; Shanghai, China, Jiangsu samples were collected from the farms of Jiangsu Wang Xianji Modern Agriculture Development Co., Ltd.; Gaoyou, China and Anhui samples were collected from the Anhui Niannian shrimp and rice cooperatives. In total, 30 samples from each location were collected for the experimental study. The tail muscles of all *P. clarkii* were sampled, fixed with anhydrous ethanol (Shanghai, China), and stored at −20 °C for later use.

### 2.2. Genomic DNA Extraction

Genomic DNA was extracted from the muscle tissue of *P. clarkii* using an Animal Genome DNA Extraction Kit (TSP201-50; Tsingke Biotechnology Co., Ltd., Shanghai, China). The specific steps were performed according to the manufacturer’s instructions. The concentration and purity were determined using a micro-ultraviolet spectrophotometer (Quawell Q5000; Thmorgan, Beijing, China) and DNA was stored at −20 °C.

### 2.3. Obtaining Microsatellite Primers and PCR Amplification

The 15 pairs of microsatellite primers were used following published studies with good amplification conditions and high polymorphism screening from Hu et al., Tan et al., and Huang et al. [21,22,23]. These primers were pre-tested and could be used as SSR primers for crayfish in this study (Table 1). Primers with a carboxyfluorescein (FAM) fluorescent marker added at the 5′ end were synthesised by Sangon Biotech Co., Ltd. (Shanghai, China).

Fifteen pairs of fluorescent primers were used to amplify the DNA of 90 samples by PCR. The reaction system comprised 25 μL 2 × Taq PCR Master Mix (including buffer, Taq DNA Polymerase, Mg^2+^, dNTPs) 12.5 μL and 1 µL of each forward and reverse primers, 0.5 μL of DNA template, and 10 μL of ddH_2_O. The PCR conditions were pre-denaturation at 94 °C for 4 min; denaturation at 94 °C for 30 s; annealing at the appropriate annealing temperature for 30 s; extension at 72 °C for 1 min, 34 cycles; post-extension for 10 min at 72 °C; and storage at 4 °C. The PCR products underwent agarose gel electrophoresis to detect the quality of the products and were then sent to Sangon Biotech Co., Ltd. for capillary electrophoresis.

### 2.4. Data Analysis

CONVERT 1.31 (Purdue University, West Lafayette, IN, USA) [24] software was used to convert the alleles into the format required for subsequent analyses. Micro-Checker v.2.2.3 (University of Hull, Hull, UK) [25] was used to check for scoring errors and detect the null alleles. PopGene 1.32 (University of Alberta, Edmonton, AB, Canada) was used to calculate the number of alleles (*N_a_*), effective allele number (*N_e_*), observed heterozygosity (*H_o_*), expected heterozygosity (*H_e_*), Shannon’s information index (*I*), gene flow (*N_m_*), Nei’s genetic distance (*D_a_*), and inbreeding coefficient for each microsatellite locus and population. The polymorphic information content (*PIC*) of each locus was calculated using PIC_CALC 0.6 (University of Pannonia, Keszthely, Hungary) [26]. The *PIC* was calculated for each population using Cervus 3.0.3 (Montana State University, Boltzmann, MT, USA) [27]. The genetic differentiation coefficient (*F_st_*) between populations was calculated using Arlequin 3.5 (University of Berne, Berne, Switzerland) [28], and the sources of variation were determined using an analysis of molecular variance (ANOVA). The Hardy–Weinberg equilibrium test was performed using the Genepop 4.7.5 (University of Montpellier, Montpellier, France). A model-based Bayesian clustering of populations of the three populations was performed using Structure 2.3.4 (University of Oxford, Oxford, UK) [29] to determine the optimal K values and analyse the genetic structure. The Admixture model with correlated allele frequencies was employed. Ten independent runs were performed for each value of K, which represents the number of genetic clusters and ranged from 1 to 8. The number of MCMC cycles was set at 10,000 iterations after a burning of equal length. The optimal K value was determined from the second-order rate of change value of the likelihood function for K (∆K) [30]. The unweighted pair group method with arithmetic mean (UPGMA) phylogenetic trees were constructed using Mega 4.0 (MS-DOX, Redmond, WA, USA) [31] based on *D_a_* values.

## 3. Results

### 3.1. Genetic Diversity of Three P. clarkii Culture Populations

Overall, a genetic diversity analysis of germplasm material from three *P. clarkii* culture populations was performed using 15 primer pairs, and the amplified bands were genotyped by high-efficiency capillary electrophoresis. In all loci, no scoring errors were found due to stuttering, and no large allele dropout was detected. All three populations had null alleles in PclG37; in addition, the CM population had null alleles in PclG8 and PclG9, the GY population had null alleles in PclG4 and PclG29, and the XC population had null alleles in PclG4, PclG8, PclG9, and PclG28. 

The *N_a_*, *N_e_*, *H_o_*, *H_e_*, *I*, and *PIC* values and mean values for 15 loci are summarised in Table 2. A total of 113 alleles were detected in the three populations, with an average of 7.5333; the *N_e_* values ranged from 1.9387 to 5.4619 with an average of 3.3147; the *H_o_* values ranged from 0.2111 to 0.9889 with an average of 0.5810; the *H_e_* values ranged from 0.4869 to 0.8215 with an average of 0.6973; the *I* values ranged from 0.8033 to 1.8259 with an average of 0.4243; and the *PIC* values ranged from 0.3670 to 0.7851 with an average of 0.6440. There were 12 loci with *PIC* values of >0.5, which were highly polymorphic and could be used for microsatellite genetic diversity analyses.

The genetic diversity of the three populations (90 samples) of *P. clarkii* is listed in Table 3. The number of alleles, number of effective alleles, observed heterozygosity, expected heterozygosity, Shannon information index, and polymorphic information content values of the XC population were the highest compared with those of the CM and GY populations, and those of the GY population were the lowest. Based on the results of each parameter, the order of genetic diversity level was as follows: Anhui Xuancheng population > Shanghai Chongming population > Jiangsu Gaoyou population. However, in general, all populations had moderate genetic diversity levels.

### 3.2. Hardy–Weinberg Equilibrium Analysis

The Hardy–Weinberg equilibrium analysis results for the three *P. clarkii* populations are presented in Table 4. The mean Hardy–Weinberg equilibrium indices of CM, GY, and XC populations were −0.1086, −0.1141, and −0.0875, respectively. Among the 15 loci in the CM population, the Hardy–Weinberg equilibrium indices of ten loci were less than 0, of which two were significantly deviated (*p* < 0.05) and five were extremely significantly deviated (*p* < 0.01). Among the fifteen GY population loci, the Hardy–Weinberg equilibrium indices of ten loci were less than 0, among which three deviated significantly (*p* < 0.05) and four deviated extremely significantly (*p* < 0.01). Among the fifteen loci of the XC population, the Hardy–Weinberg equilibrium indices of nine loci were less than 0, of which two deviated significantly (*p* < 0.05) and five deviated extremely significantly (*p* < 0.01).

### 3.3. Genetic Structure and Differentiation

The statistics of inbreeding coefficient *(F_is_*), total inbreeding coefficient (*F_it_*), inter-population genetic differentiation coefficient (*F_st_*), and gene flow (*N_m_*) of *P. clarkii* populations showed that *F_is_* was negative in five loci (PcLG02, PcLG07, PcLG13, PcLG15, and PcLG33) and the rest were positive, with an average value of 0.1001. *F_it_* was negative for PcLG02, PcLG13, PcLG15, and PcLG33, and positive for the rest, with an average value of 0.1623. The *F_st_* values of the 15 sites ranged from 0.0079 to 0.1547, with an average value of 0.0691. The gene flow (*N_m_*) at each site ranged from 1.3662 to 31.3790, with an average of 3.3658 (Table 5).

The calculations of Nei’s genetic distance and Wright’s F-statistics for the three populations showed that the genetic distance was largest between CM and GY (*D* = 0.2560) and smallest between CM and XC populations (*D* = 0.1602) and, overall, the genetic distances were <0.3 (Table 6). The *F*-statistic was largest between CM and GY (*F_st_* = 0.1068) and smallest between CM and XC *(F_st_* = 0.0553) and, overall, the genetic differentiation index was higher than 0.05 and lower than 0.15, indicating that the degree of genetic differentiation among the populations was moderate. The ANOVA results (Table 7) showed that 91.51% of the genetic variation came from intra-population variation and 8.49% from inter-population variation, and inter-population genetic differentiation reached a highly significant level (*p* < 0.01).

**Figure 1:**
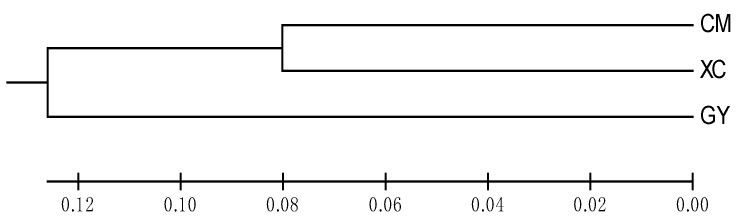
Unweighted pair group method with arithmetic mean clustering tree of the three *P. clarkii* stocks based on Nei’s genetic distance. The groups of crayfish from three geographical regions (CM = Shanghai Chongming, GY = Jiangsu Gaoyou, XC = Anhui Xuancheng).

The UPGMA cluster tree constructed based on *D_a_* values is shown in Figure 1. The CM and XC populations were clustered into one branch, whereas the GY population was clustered alone.

**Figure 2:**
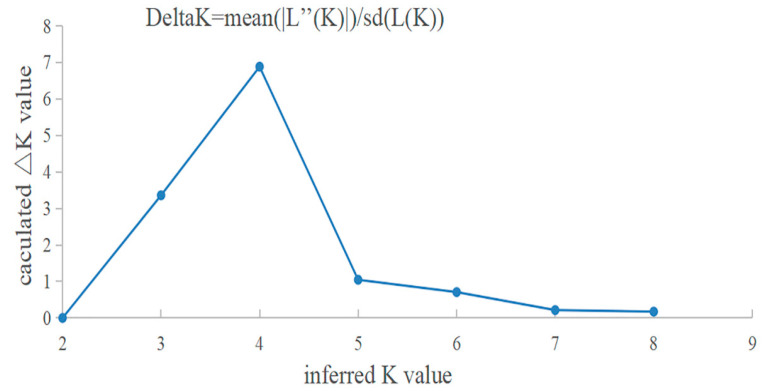
Curve of change in Delta K with changing K values.

**Figure 3:**
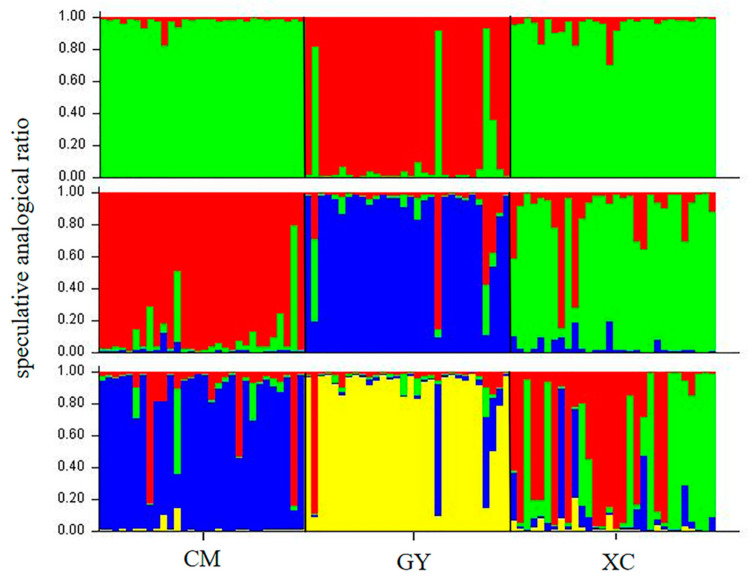
Bayesian clustering of *P. clarkii* cultured individuals. Bar plots show the individual membership coefficients at K = 2–4, where K is the number of inferred genetic clusters in the model. CM = Shanghai Chongming, GY = Jiangsu Gaoyou, XC = Anhui Xuancheng.

Figure 2 shows the trend curves of DeltaK values and different hypothetic K values. When K = 4, a peak was found, indicating that all sample individuals could be divided into four groups. Figure 3 shows the genetic structures for K = 2, K = 3, and K = 4. When K = 2, all individuals of CM and XC are grouped into the same genetic cluster, and most individuals of GY are grouped into another genetic cluster, which corresponds to the results of the UPGMA tree. Notably, three individuals of GY were assigned to the same genetic cluster as CM and XC, in addition to one individual who was admixed. When K = 3, we can clearly distinguish the genetic clustering of the three main corresponding sampled populations. When K = 4, it can be seen that the XC population has a higher genetic heterogeneity than the CM and GY populations.

## 4. Discussion

### 4.1. Genetic Diversity of Microsatellite Loci and Cultured Populations of P. clarkii

The inbreeding of *P. clarkii* has resulted in a loss of various group genes and a degeneration of the germplasm [19]. Genetic diversity forms the basis of species diversity. It is generally believed that the higher the genetic diversity of a species, the stronger its ability to adapt to the environment [18]. Genetic assessments are conducted to investigate genetic diversity, which is important for understanding the evolution and adaptability of species [32,33,34]. Therefore, investigating the genetic diversity of *P. clarkia* is important for germplasm conservation and genetic breeding.

The higher the *N_e_*, *H_e_*, and *PIC* values of the genetic diversity parameters, the richer the population’s genetic diversity [35]. In general, populations with a low genetic diversity are less resilient to changing environments than their peers [36]; thus, it is easier for them to be eliminated. It is generally believed that the *PIC* value of high polymorphism is greater than 0.5, the *PIC* value of moderate polymorphism is between 0.25 and 0.5, and a *PIC* value of lower than 0.25 indicates a low polymorphism, which is not suitable for breeding [37,38,39]. Among the fifteen microsatellite loci selected in this study, three were moderately polymorphic and twelve were highly polymorphic. The average *PIC* was 0.6640, which was higher than those reported by Xing et al. [20] and Tan et al. [22]. The effective allele number is also an important parameter of genetic diversity. The average effective allele number of the 15 loci selected in this study was 3.3147, which was higher than that reported by Huang et al. [23] in a study of *P. clarkii* in the Guangxi region, but similar to that reported in a study by Xing et al. [20] on *P. clarkii* in the Jiangsu region. Gene heterozygosity is the most suitable parameter for measuring population genetic diversity [40] and can be divided into expected heterozygosity and observed heterozygosity. The higher the heterozygosity, the higher the genetic diversity within the population; otherwise, the smaller the genetic diversity within the population [41]. When the values are similar, it means that factors such as external environmental selection and inbreeding did not have a significant effect on them [42]. In this study, we determined that the average expected heterozygosity of the three populations of *P. clarkii* was 0.5810 and the average observed heterozygosity was 0.6973, which was higher than the expected heterozygosity. Further analyses of heterozygosity among the three populations showed that the heterozygosity of the Xuancheng population was the highest, the Chongming population was in the middle, and the Gaoyou population was the lowest. In summary, the Xuancheng population has a higher genetic diversity and a better genetic background. In our opinion, this may be related to geographical location and production. Chongming, Shanghai, is an island connected to Shanghai city by a bridge, and there is lower exchange of populations with other regions. Gaoyou, Jiangsu, has a lower production of crayfish culture but is rich in Chinese mitten crabs, while Xuancheng, Anhui, is the main production area of crayfish culture, so it may have more geographic populations. Beyond that, Chang et al. studied the genetic diversity of *Eriocheir sinensis* and found that samples collected a had high genetic diversity, probably because when female eriocheir crabs become sexually mature, they tend to attract several male *Eriocheir sinensis* to donate sperm to their sperm storage chambers [43]. *P. clarkii* has a similar mating pattern, in which a single female tends to mate with multiple males, which facilitates increased genetic diversity. Fitzsimmons also believes that multiple paternities can increase genetic diversity by increasing genetic heterozygosity, which provides a good mechanism to reduce inbreeding [44]. In summary, genetic diversity varies among populations and it is known that there are no significant differences in genetic parameters between populations in similar areas, so distant crosses should be made between individuals to ensure the stability of genetic diversity [16]. However, it is worth noting that crosses should be performed in a relatively closed culture environment to avoid widespread genetic contamination. In this study, we mainly compared the genetic diversity of three *P. clarkii* cultured populations, and the results were not sufficient to prove that one or a few microsatellite loci could be used for the identification of the origin of *P. clarkii*. Certainly, the identification of population origin is a very interesting matter, which still requires a large amount of relevant studies, at least with more loci and more accessions of the population. Morphological traits may be a tool to identify the origin of populations because of their ease of use. Therefore, studying the relationship between phenotypic traits and genes may be a better approach. 

The Hardy–Weinberg equilibrium index reflects the equilibrium relationship between *H_o_* and *H_e_*. The closer the value of the Hardy–Weinberg equilibrium index is to 0, the more the the genotype distribution tends to be balanced. Among the 45 genetic seats in the three populations and fifteen microsatellite loci in this study, seven genetic loci in each population deviated from the Hardy–Weinberg equilibrium. Among them, all three populations had six genetic loci exhibiting deviation from the Hardy–Weinberg equilibrium due to heterozygote deficiency (*D* < 0, *F_is_* > 0), while the Chongming and Xuancheng populations had one deviation from the equilibrium due to heterozygote excess (*D* > 0, *F_is_* < 0) and the Gaoyou population had one genetic loci deviating from equilibrium due to other reasons (*D* > 0, *F_is_* > 0) [45,46]. The mean value of the Hardy–Weinberg equilibrium index showed that the Xuancheng population deviated relatively little and the Gaoyou population deviated relatively largely. The heterozygote deficiency phenomenon may be due to limited study samples and inbreeding, and the heterozygote excess phenomenon may be due to frequent gene exchange between the study subjects and other populations, such as the artificial introduction of exotic individuals carrying different alleles [47,48,49]. However, both phenomena rarely occur in large and stable populations [50]. The presence of both a heterozygous deficiency and a heterozygous excess in the Chongming and Xuancheng populations in this study suggests that they may have both been inbred and experienced distant introductions, but since the mean Hardy–Weinberg equilibrium was negative, this suggests that the former showed a greater impact. Crayfish have a high reproductive capacity and are highly susceptible to mating of inbred individuals during culture, which can be detrimental in the long run, hence the need to enhance mating in populations from different regions to improve inbreeding. From the results of this study, the relatively low level of inbreeding in the Xuancheng population indicates that its fry are of the best quality. We speculate that it is possible that the negative effects of inbreeding are weakened by introgression. In recent years, increasingly more farmers have focused on introducing crayfish populations from long distances, which has enhanced the possibility of genetic variation, improved the genetic diversity of the population, and slowed down the degradation of the germplasm caused by self-propagation and inbreeding. Several studies [51] have suggested that hybridisation can increase the genetic heterozygosity and genetic diversity. Liang et al. [51] suggested that hybridisation between two populations increased genetic diversity indicators such as *N_a_*, *H_e_*, and *H_o_* in oysters and significantly reduced *F_is_*. Hu et al. [52] suggested that intraspecific hybridisation can cause recombination of existing genes, which is an important way to increase biodiversity. However, genetic pollution is indeed a noteworthy problem, which reminds us that we cannot cross randomly, but must develop appropriate crossbreeding strategies to avoid genetic pollution. In addition, before successfully selecting new populations, the selected population should be strictly separated from the outside population to avoid the spread of foreign genes.

*P. clarkii* is a highly invasive species that reproduces rapidly, and coupled with the extensive farming activities and the negative effects of inbreeding, among others, of *P. clarkia*, their numbers will significantly expand in the absence of any measures. As shown in the previous results, there are certain differences in genetic diversity between different populations. Therefore, a more scientific breeding program should be developed to maintain the level of genetic diversity; however, such a breeding program is currently not existed. The establishment of a breeding program needs to be supported by a large number of studies, including more loci and as full a range of populations as possible, and, if possible, private alleles of different populations should also be found to establish a breeding program.

### 4.2. Genetic Structure and Differentiation of the Three Cultured P. clarkii Populations

The genetic differentiation index is an important parameter reflecting the degree of genetic differentiation between populations, which is measured by alleles at microsatellite loci [53]. The results of the ANOVA showed that genetic variation was mainly derived from within the population (91.51%), and inter-population genetic variation accounted for 8.49%. In this study, the genetic differentiation index among populations was medium, with the lowest being between the Xuancheng population and the Chongming population, and the highest being between the Gaoyou population and the Chongming population. The average total *N_m_* level of each population was 3.3658, and the *N_m_* level among all populations was moderate, except for the Xuancheng and Chongming populations, which were slightly higher than 4.0. In general, there was a certain degree of genetic differentiation between the three populations, among which the Xuanxeng population and Chongming population had a higher gene flow and a lower genetic differentiation index, indicating that gene exchange occurred between the two populations. However, the distance between the two populations was relatively long, and Chongming was an island, so we inferred that the gene exchange between the two populations was caused by human factors. The results of the genetic diversity analysis also showed that the genetic diversity of the Chongming population was higher than that of the Gaoyou population but lower than that of the Xuancheng population. It is almost impossible for crayfish to reach the Chongming area through natural migration, indicating that crayfish in the Chongming area had been introduced. Rumishaa et al. found that the gene flow among sawn crab populations was large enough to offset population homogeneity [54]. Additionally, previous studies have revealed that higher gene flow levels can resist germplasm decay caused by genetic drift [55]. Therefore, seedlings from the Chongming and Xuancheng populations with higher gene flow levels may be of higher quality and therefore have higher breeding potential. *F_is_* is an important parameter for evaluating the degree of inbreeding between individuals, and generally refers to the probability that two identical genes of an individual come from the same ancestor. A positive *F_is_* within a population indicates obvious inbreeding within that population [18,56,57,58]. In this study, the average *F_is_* value of each locus was 0.1001, indicating that each population had a certain degree of inbreeding. Inbreeding is inevitable in aquaculture, and we should establish specific breeding programs based on the breeding characteristics in cultured species to try to avoid a large degree of inbreeding.

In this study, the minimum genetic distance (0.1602) was found between the Xuacheng population and the Chongming population, and the maximum genetic distance (0.2560) was identified between the Chongming and Gaoyou populations. The UPGMA phylogenetic tree showed that the Xuancheng and Gaoyou populations first clustered together, indicating that they were closely related, which is consistent with the results of genetic differentiation. There may be a possibility of mutual introduction between the Xuancheng and Chongming groups. Due to the interference of human factors, there was no absolute correlation between geographical distance and genetic distance. For example, in this study, the geographical distances between the three sites were close to each other, similar to an equilateral triangle; however, the genetic distance between the two differed.

From the genetic structure diagram, when K = 2, the structure analysis results were consistent with the UPGMA cluster tree constructed based on *D* values. The two populations of Chongming and Xuancheng were grouped together, indicating that they have some genetic homology. This may be because the initial populations of the two populations are closely related; however, they have been cultured in different environments and gradually localised to form different populations. When K = 3, the Chongming and Xuancheng populations also had a certain genetic similarity; however, when K = 4, the Xuancheng population was clearly different from the other two populations, which could be partly due to the increased mixing of other geographic groups.

Overall, all three crayfish cultured populations are potential genetic resources in China and should be protected. It is recommended that a genetic resource bank should be established to register the crayfish populations to more deeply understand genetic information, which will help in the development of breeding programs.

## 5. Conclusions

Genetic diversity includes the level of variation and the distribution pattern of variation, namely the genetic structure of populations, and is an important theoretical basis for biodiversity conservation [59]. The high genetic diversity of the three regional *P. clarkii* breeding populations may be due to different regional introductions, with the Xuancheng population in Anhui having the highest genetic diversity. There was some genetic differentiation and germplasm mixing among various populations. The Shanghai Chongming and Anhui Xuancheng populations are closely related and may have mutual genetic introgression. Overall, the Anhui Xuancheng population had the highest genetic diversity and a relatively more diverse genetic structure, mixing individuals from multiple geographic groups. If the Anhui population is used as the base population for genetic breeding, there will be a higher chance of obtaining high-quality genes, and therefore the breeding efficiency can be improved. Although the population selected for this study was a high yielding area in China, the number was low and a wider group should be included in future studies. In this study, a genetic evaluation of three *P. clarkii* breeding populations was conducted to provide a reference for population selection in breeding efforts and to suggest a suitable breeding program to maintain genetic diversity and conserve genetic resources.

## Figures and Tables

**Table 1:** Information on 15 pairs of microsatellite primers for *P. clarkii*.

Locus	Primer Sequences	Repeat Motif	Annealing Temperature (°C)	Size (bp)
PcLG02	F:CTCCCCATGCACTCTGGCTCTGT R:TGGCGAATTTTGCCTGTTTCTGTC	(GATA)_3_GAGAA(GATA)_5_	66	216–224
PcLG03	F:CTCTCCACCAGTCATTTCTT R:AAGCTTACAATAAATATAGATAGAC	(TCTA)_20_	52	216–420
PcLG04	F:TATATCAGTCAATCTGTCCAG R:TCAGTAAGTAGATTGATAGAAGG	(TCTA)_3_...(TCTA)_2_...(TCAT)_29_...(TCTA)_2_	54	170–290
PcLG07	F:CCTCCCACCAGGGTTATCTATTCA R:GTGGGTGTGGCGCTCTTGTT	(TCTA)_8_	63	100–160
PcLG08	F:ACGATAAATGGATAGATGGATGAA R:CCGGGTCTGTCTGTCTGTCA	(GATA)_16_	62	148–220
PcLG09	F:TATGCACCTTTACCTGAAT R:TGTTGGTGTGGTCATCA	(TCTA)_14_	60	80–160
PcLG13	F:CTCTCCTGGCGCTGTTATTTAGC R:TGAAGAGGCAGAGTGAGGATTCTC	(TCTA)_12_	62	130–150
PcLG15	F:GGCGTGACGCCAACGTGTCTT R:GGCTGGCCACTTTGTTAGCCTGAG	(TATC)_2_TGTC(TATC)_17_TATT(TATC)_3_	70	150–185
PcLG16	F:CTCGGAATGTCCACCTGAGA R:TCATTATGGATTTTGTCAATCTAT	(TCTA)_18_TCTC(TATC)_3_	54	80–160
PcLG17	F:GTCGGGAACCTATTTACAGTGTAT R:AAGAGCGAAGAAAGAGATAAAGAT	(TCTA)_14_	57	156–190
PcLG27	F:AATCTTAAGATCATGAAAAAGGTA R:TTTAAGGAACGTATAAGAAAAGAC	(TATC)_4_CATC(TATC)_8_	57	80–150
PcLG28	F:CTCGGCGAGTTTACTGAAAT R:AGAAGAAAGGGATATAAGGTAAAG	(GATA)_22_(GA)_5_	60	210–270
PcLG29	F:GAAAGTCATGGGTGTAGGTGTAAC R:TTTTTGGGCTATGTGACGAG	(TATC)_9_	65	95–165
PcLG33	F:TTCGAGGCGTTGCTGATTGTAAGT R:CAAGGAAGCGTATAGCCGGAGTCT	(GT)_21_	68	120–180
PcLG37	F:TAAATAAGTGGCGTGTAAGACGAG R:TAACTAAGCCAGGGTGGTCTCCAG	(CA)_4_CG(CA)_15_CG(CA)_23_	66	80–180

**Table 2:** Genetic diversity parameters among 15 loci in *P. clarkii*.

Locus	Sample Size	*N_a_*	*N_e_*	*H_o_*	*H_e_*	*I*	*PIC*
PclG02	180	3	2.0436	0.6333	0.5135	0.8033	0.4142
PclG03	180	7	2.1141	0.4111	0.5299	0.9818	0.4643
PclG04	180	7	2.7825	0.3222	0.6442	1.2597	0.5821
PclG07	180	8	2.8531	0.6444	0.6531	1.3044	0.5906
PclG08	180	10	3.9589	0.5000	0.7516	1.5920	0.7109
PclG09	180	7	4.4690	0.3778	0.7806	1.6326	0.7412
PclG13	180	2	1.9387	0.5333	0.4869	0.6773	0.3670
PclG15	180	8	3.6594	0.9333	0.7308	1.5382	0.6917
PclG16	180	8	5.2427	0.6556	0.8138	1.8259	0.7851
PclG17	180	8	3.3109	0.5111	0.7019	1.3933	0.6465
PclG27	178	9	4.7374	0.6966	0.7934	1.7041	0.7573
PclG28	178	8	3.8303	0.6067	0.7431	1.5229	0.6988
PclG29	180	7	5.4619	0.6889	0.8215	1.7646	0.7917
PclG33	180	10	3.6751	0.9889	0.7320	1.5846	0.6906
PclG37	180	11	4.1422	0.2111	0.7628	1.7496	0.7275
mean	180	7.5333	3.3147	0.5810	0.6973	1.4243	0.6440

Note: *N_a_* = number of different alleles; *N_e_* = number of effective alleles; *H_o_* = observed heterozygosity; *H_e_* = expected heterozygosity; *I* = Shannon’s information index; *PIC* = polymorphic information content.

**Table 3:** Summary of genetic diversity of the three *P. clarkii* stocks.

Population	Sample Size	*N_a_*	*N_e_*	*I*	*H_o_*	*H_e_*	*PIC*
CM	60	5.2667	3.0448	1.2329	0.5600	0.6440	0.5774
GY	60	4.8000	2.7969	1.1292	0.5567	0.6166	0.5446
XC	60	6.2000	3.5470	1.3987	0.6257	0.7086	0.6452
mean	60	5.4222	3.1296	1.2536	1.7424	0.6564	0.5891

Note: *N_a_* = number of different alleles; *N_e_* = number of effective alleles; *H_o_* = observed heterozygosity; *H_e_* = expected heterozygosity; *I* = Shannon’s information index; *PIC* = polymorphic information content. The groups of *P. clarkii* from three geographical regions (CM = Shanghai Chongming, GY = Jiangsu Gaoyou, XC = Anhui Xuancheng).

**Table 4:** Analysis of Hardy–Weinberg equilibrium at 15 loci among different populations of *P. clarkii*.

Locus	CM	GY	XC
PclG02	0.1785	0.0330	0.6991
PclG03	−0.1709 *	−0.3022	−0.2027
PclG04	−0.0436 *	−0.4306 **	−0.7793 **
PclG07	−0.1529	0.1689	0.0492 *
PclG08	−0.4788 **	−0.1991 *	−0.2916 **
PclG09	−0.6903 **	−0.1618 **	−0.5092 **
PclG13	0.2248	−0.0068	0.0679
PclG15	0.1164	0.3755	0.3949
PclG16	−0.1054	−0.1213 *	−0.1883
PclG17	−0.2058	−0.2387	−0.0295 *
PclG27	−0.1360	−0.1904	0.1149
PclG28	−0.1748 **	0.0146 *	−0.2669 **
PclG29	0.0328	−0.3306 **	−0.0702
PclG33	0.7167 **	0.2647	0.4148
PclG37	−0.7395 **	−0.5864 **	−0.7150 **
mean	−0.1086	−0.1141	−0.0875

Note: * indicates statistical significance (*p* < 0.05); ** indicates statistical significance (*p* < 0.01). The groups of *P. clarkii* from three geographical regions (CM = Shanghai Chongming, GY = Jiangsu Gaoyou, XC = Anhui Xuancheng).

**Table 5:** *F*-statistics and gene flow of 15 microsatellite loci in *P. clarkii*.

Locus	Sample Size	*F_is_*	*F_it_*	*F_st_*	*N_m_*
PclG02	180	−0.3014	−0.2402	0.0470	5.0668
PclG03	180	0.2060	0.2199	0.0175	14.0738
PclG04	180	0.4481	0.4970	0.0886	2.5732
PclG07	180	−0.0296	0.0078	0.0363	6.6361
PclG08	180	0.3030	0.3310	0.0401	5.9784
PclG09	180	0.4352	0.5133	0.1383	1.5578
PclG13	180	−0.1103	−0.1015	0.0079	31.3790
PclG15	180	−0.3111	−0.2843	0.0205	11.9627
PclG16	180	0.1246	0.1899	0.0746	3.1012
PclG17	180	0.1337	0.2677	0.1547	1.3662
PclG27	178	0.0480	0.1185	0.0741	3.1260
PclG28	178	0.1344	0.1799	0.0526	4.5057
PclG29	180	0.1027	0.1567	0.0601	3.9064
PclG33	180	−0.4691	−0.3585	0.0752	3.0736
PclG37	180	0.6889	0.7217	0.1055	2.1187
mean	180	0.1001	0.1623	0.0691	3.3658

Note: *F_is_* = inbreeding coefficient related to subpopulations; *F_it_* = inbreeding coefficient related to total population; *F_st_* = genetic differentiation index; *N_m_* = gene flow.

**Table 6:** Nei’s genetic distance (below diagonal line), Wright’s *F*-statistics (above diagonal line), and gene flow (in parentheses) of the three *P. clarkii* stocks.

Population	Chongming	Gaoyou	Xuancheng
Chongming	-	0.1068 (2.0908)	0.0553 (4.2708)
Gaoyou	0.2560	-	0.0927 (2.4469)
Xuancheng	0.1602	0.2478	-

Note: The significant *p* value for each pairwise genetic distance was less than 0.01.

**Table 7:** ANOVA analysis of genetic structure of *P. clarkii* stocks.

Source of Variation	Degree of Freedom (*d.f.*)	Sum of Squares	Variance Components	Percentage of Variation	*F*-Statistic (*F_st_*)	*p*-Value
Among population	2	64.433	0.45516	8.49		
Within population	177	868.600	4.90734	91.51		
Total	179	933.033	5.36250		0.08488	0.000 **

Note: ** indicates statistical significance (*p* < 0.01).

## Data Availability

The data analysed for this study are available from the corresponding author upon reasonable request.
